# Perceived Access to Endometriosis Care Among Hispanic Women: A Cross-Sectional Survey

**DOI:** 10.7759/cureus.113230

**Published:** 2026-07-23

**Authors:** Eliana M Burgos, Courtney Chalmers, Jean P Tanner, Jason Salemi, Emad Mikhail, Courtney Mascoe, Diana Encalada-Soto

**Affiliations:** 1 Medicine, University of South Florida Morsani College of Medicine, Tampa, USA; 2 Epidemiology, University of South Florida College of Public Health, Tampa, USA; 3 Obstetrics and Gynecology, University of South Florida Morsani College of Medicine, Tampa, USA

**Keywords:** access to health care, endometriosis, hispanic population, perceived barriers, women’s health

## Abstract

Primary objective

We evaluated perceived access to endometriosis-related care among Hispanic and non-Hispanic women with endometriosis.

Secondary objectives

We compared referral experiences, financial barriers, geographic access, and specialist access between Hispanic and non-Hispanic women and between women residing within and outside of the United States.

Design

This was a cross-sectional, anonymous, web-based survey.

Setting

This was an international, online survey including participants in and outside of the United States.

Participants

A total of 288 adults aged ≥18 years with suspected or confirmed endometriosis, fluent in English or Spanish, were recruited in the study.

Interventions

None.

Measurements and main results

The primary outcome was perceived access to care. Across all groups, fewer than one-third of respondents reported adequate access to knowledgeable endometriosis clinicians, with no significant differences observed when comparing U.S. Hispanic (p=0.99) or non-U.S. Hispanic (p=0.10) participants to the U.S. non-Hispanic reference group. Secondary outcomes revealed significant differences in access-related experiences between groups. Hispanic participants were less likely to have been evaluated by an endometriosis specialist when comparing non-U.S. Hispanics and U.S. non-Hispanics only: 47 (55%) vs. 92 (75%), respectively (p=0.002). Diagnostic delays were common, with most participants reporting more than five years between symptom onset and diagnosis. Although not significant, approximately one-third or more of participants across groups reported consulting more than five providers before diagnosis (9 (32%) U.S. Hispanics to 33 (65%) non-U.S. non-Hispanics). Difficulty obtaining referrals was more commonly reported among Hispanic respondents, as seen in 17 (61%) U.S. Hispanics and 68 (79%) non-U.S. Hispanics reporting difficulty compared to 49 (40%) U.S. non-Hispanics (p=0.04 and p<0.001, respectively). Non-U.S. Hispanics reported greater financial barriers, 60 (70%) non-U.S. Hispanics to 65 (53%) U.S. non-Hispanics (p=0.01). Geographic access varied: U.S. Hispanics were less likely to require out-of-state travel, 2 (7%) U.S. Hispanics to 32 (26%) U.S. non-Hispanics (p=0.04), whereas non-U.S. Hispanics more often required cross-border travel, with 34 (40%) to 32 (26%) U.S. non-Hispanics (p=0.04).

Conclusion

Hispanic respondents in this survey, particularly those living outside the US, reported substantial barriers to endometriosis care, including limited specialist access, referral challenges, financial strain, and travel burden. These findings suggest potential targets for future interventions, including referral pathways, specialist availability, and patient navigation, which warrant further evaluation.

## Introduction

Endometriosis is a chronic, inflammatory disease affecting an estimated 10% of reproductive-age women worldwide [1]. It is estrogen-based and associated with infertility, pelvic pain, and diminished quality of life [2,3]. Diagnosis is often delayed by 7-12 years after symptom onset, contributing to worsening physical, emotional, and social outcomes [3,4]. Diagnostic delay has been consistently attributed to symptom normalization, dismissal of pain complaints, and variability in presenting symptoms [5-7]. Beyond its clinical burden, endometriosis imposes significant quality of life burdens and economic costs, with estimates exceeding $69 billion annually in the United States (US) due to both medical costs and loss of work productivity among affected women [3,8].

Growing evidence indicates that the burden of endometriosis and access to effective care are not experienced equally across populations. Racial, ethnic, and socioeconomic disparities contribute to inequities in diagnosis, management, and surgical outcomes [2,9]. Historically believed to primarily affect White women, endometriosis is now recognized as similarly or more prevalent among Hispanic, Black, and Asian women, yet inequities in care persist [2,9]. For example, Hispanic and Black women are less likely than White women to undergo minimally invasive surgery and more likely to experience perioperative complications [9], reflecting systemic differences in referral patterns, insurance coverage, and access to specialists [3].

Hispanic patients with endometriosis appear to face particularly pronounced barriers to obtaining comprehensive care possibly due to differences in pain expression and cultural norms [10,11]. These barriers persist despite many Hispanic respondents being educated and employed, suggesting structural rather than individual-level factors. Additional studies show that women from minority racial and ethnic groups in the US are more likely to encounter misdiagnosis, stigmatization, and dismissal of pain complaints when seeking care for endometriosis-related symptoms [3,12].

Younger women and women from racial and ethnic minority groups consistently report lower satisfaction with their endometriosis care and reduced perceptions of patient-centeredness [4]. Conceptual frameworks such as the Perceived Access to Healthcare Questionnaire (PAHQ) emphasize that meaningful access extends beyond availability and affordability to include acceptability, accommodation, and awareness [13]. These dimensions are particularly relevant for Hispanic patients, who may face compounded language, cultural, and geographic barriers.

Despite the high prevalence of endometriosis, the disproportionate challenges experienced by marginalized populations, and persistent gaps in equitable care, little is known about how Hispanic women, particularly those living outside the US, perceive access to endometriosis care across referral, financial, and geographic domains. To address this gap, we conducted an international, web-based survey to examine perceived access to care and patient-centeredness among women with endometriosis. The primary objective was to assess perceived access to care across groups defined by Hispanic ethnicity and country of residence. Secondary objectives were to compare referral experiences, specialist access, financial barriers, and geographic access between groups to identify barriers that may inform more culturally responsive and equitable models of endometriosis care.

## Materials and methods

We conducted a cross-sectional study using an anonymous, open, web-based survey administered through Qualtrics, a secure online survey platform, to assess perceived access to endometriosis-related care and associated barriers. Exact survey questions can be found in the Appendices. This study was reviewed by the University of South Florida Institutional Review Board and determined to be exempt from full review (IRB study #007633). Participants provided electronic informed consent before survey initiation. Data were collected between January 29, 2025, and April 10, 2025.

Eligible participants were adults aged ≥18 years who were fluent in English or Spanish and who self-reported a previous clinical diagnosis or suspicion of endometriosis. The majority reported physician-confirmed diagnoses. Individuals who did not meet age, diagnostic, or language criteria were excluded. The survey was open internationally, and the country of residence was self-reported.

Participants were recruited through convenience sampling using an open survey design. Recruitment occurred via internet-based outreach and community-based advertising, including social media platforms, national and international endometriosis advocacy organizations, and printed flyers posted in outpatient obstetrics and gynecology clinics at the study institution. Recruitment materials invited individuals with suspected or confirmed endometriosis to voluntarily participate in a brief survey about access to endometriosis-related care. Participation was not required to access any website, service, or clinical care, and no incentives were offered.

The survey was administered entirely online, and responses were captured automatically by the Qualtrics platform without manual data entry. Only fully completed questionnaires were included in the analytic sample. Because recruitment occurred through an open survey design distributed across multiple online and community-based platforms, view, participation, and completion rates could not be reliably calculated.

The electronic informed consent page described the study’s purpose, investigator identities, the voluntary nature of participation, the estimated completion time (approximately 5 minutes), the types of data collected, and data storage procedures. Participants were informed that no personally identifiable information would be collected and that study data would be retained for five years following study completion.

Survey data were stored on password-protected Qualtrics servers and downloaded to encrypted, institutionally approved systems accessible only to authorized study personnel. Cookies were used by the Qualtrics platform to reduce the likelihood of multiple survey submissions. IP address checks, log file analyses, and timing-based exclusions were not performed.

The questionnaire was available in English and Spanish and included items assessing demographics, clinical history, and perceived access to endometriosis-related care. Survey items were adapted from existing instruments, including the PAHQ and the World Endometriosis Research Foundation’s EPHect Patient Questionnaire. Because the final instrument incorporated adapted and newly developed items, it was intended to assess feasibility and clarity rather than serve as a formal validation study.

Before dissemination, the survey underwent internal testing by the research team to assess usability, readability, and technical functionality within the Qualtrics platform. Spanish-language translation accuracy was confirmed through back-translation and review by bilingual researchers to ensure linguistic and cultural equivalence. Survey items were presented in a fixed order with adaptive questioning for certain items. The questionnaire consisted of approximately 10 items distributed across five screens. Missing responses were permitted, and completeness was assessed post-hoc. Respondents were able to review and modify their responses before final submission. Finally, because the survey was anonymous and contained demographic and geographic detail that could pose a re-identification risk for small subgroups, the de-identified dataset and analysis code are available from the corresponding author on reasonable request, subject to confirmation that re-identification risk is minimized.

The primary outcome was perceived access to endometriosis-related care. Secondary outcomes included access to endometriosis specialists, referral experiences, financial barriers, travel burden, and other reported barriers to obtaining care.

Quantitative analyses were descriptive and comparative. Categorical variables are summarized using frequencies and percentages. Participants were stratified by Hispanic ethnicity and country of residence into four groups: U.S. non-Hispanic, U.S. Hispanic, non-U.S. non-Hispanic, and non-U.S. Hispanic. Chi-square or Fisher’s exact test was used for categorical comparisons. Pairwise comparisons used U.S. non-Hispanic participants as the reference group. Analyses were exploratory, and no statistical weighting or correction was applied. A two-sided p-value <0.05 was considered statistically significant.

## Results

Sample characteristics

**Figure 1:**
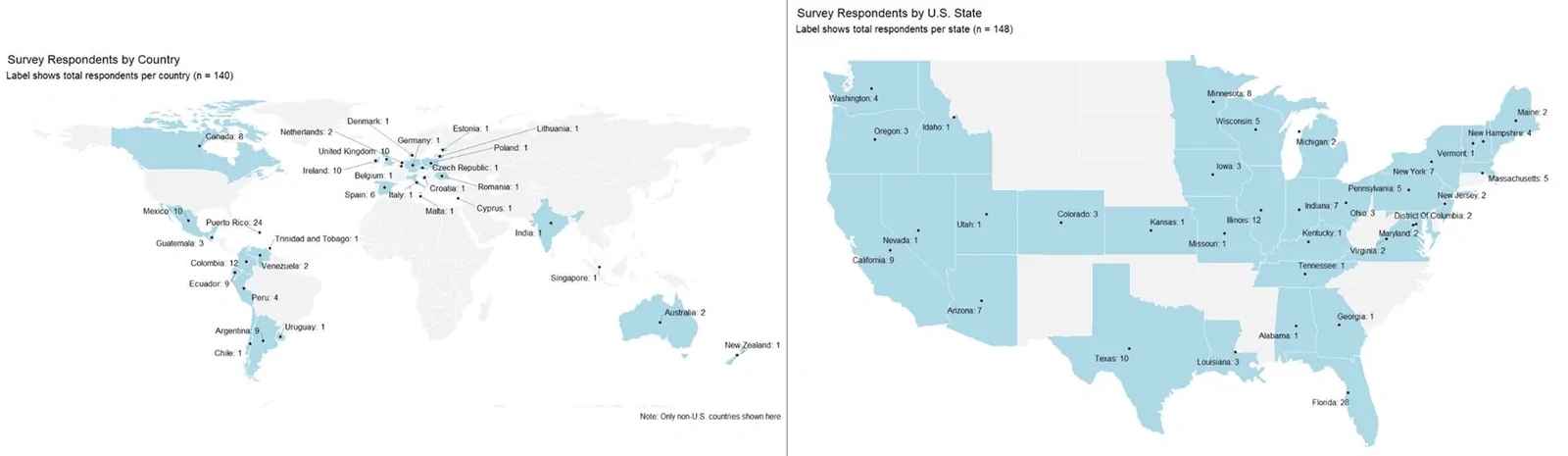
Survey Respondents by Country and U.S. State No artificial intelligence (AI) tools were used in the creation, design, or modification of the figure.

Among the 288 participants who completed the survey and were included in the analysis, 151 resided in the US and 137 outside the US, with notable geographic diversity across both Hispanic and non-Hispanic respondents (Figure 1).

Sociodemographic differences across groups were substantial, as noted in Table 1. U.S. Hispanic participants had a significantly different age distribution and were more racially diverse than U.S. non-Hispanics, while non-U.S. Hispanic participants were significantly older and reported lower educational attainment. Employment patterns differed mainly among non-U.S. non-Hispanic respondents, who were more likely to be students.

**Table 1:** Sociodemographic Characteristics Among Survey Participants by Ethnicity and Country of Residence Chi-square or Fisher’s exact test was used to assess differences across the four ethnicity/country of residence groups. *Represents differences across the four ethnicity/country of residence groups. †Pairwise comparisons were conducted between each group and U.S. non-Hispanic participants (categorical variables). ‡χ² statistics are shown, where χ² tests were used. For comparisons with sparse cell counts, Fisher’s exact test was applied; a χ² statistic is therefore not reported for those comparisons. P-values<0.05 considered statistically significant. U.S.: United States; χ² = chi-square; df: degrees of freedom; NA: not applicable

		U.S. residents				Non-U.S. residents							
Characteristics	All (n=288)	Non-Hispanic (n=123)	Hispanic (n=28)	p-value†	Test statistic‡	Non-Hispanic (n=51)	p-value†	Test statistic‡	Hispanic (n=86)	p-value†	Test statistic‡	p-value*	Test statistic‡
Age group				0.002	NA		0.89	NA		<0.001	χ²=146.51, df=4	<0.001	χ²=184.59, df=12
18-25	49 (17.0)	24 (19.5)	7 (25.0)			8 (15.7)			10 (11.6)				
26-35	84 (29.2)	50 (40.7)	9 (32.1)			24 (47.1)			1 (1.2)				
36-45	63 (21.9)	42 (34.1)	4 (14.3)			16 (31.4)			1 (1.2)				
46-55	49 (17.0)	6 (4.9)	4 (14.3)			3 (5.9)			36 (41.9)				
>55	43 (14.9)	1 (0.8)	4 (14.3)			0			38 (44.2)				
Race				<0.001	NA		0.02	χ²=5.60, df=1		<0.001	χ²=30.40, df=1	<0.001	χ²=34.07, df=3
White	225 (78.1)	114 (92.7)	17 (60.7)			41 (80.4)			53 (61.6)				
Other	63 (21.9)	9 (7.3)	11 (39.3)			10 (19.6)			33 (38.4)				
Highest education level				0.74	NA		0.38	χ²=1.92, df=2		0.005	χ²=10.59, df=2	0.008	χ²=17.44, df=6
HS or less	45 (15.6)	13 (10.6)	3 (10.7)			5 (9.8)			24 (27.9)				
Some college	44 (15.3)	22 (17.9)	3 (10.7)			5 (9.8)			14 (16.3)				
College graduate	199 (69.1)	88 (71.5)	22 (78.6)			41 (80.4)			48 (55.8)				
Employment status				0.21	NA		0.005	χ²=10.49, df=2		0.44	χ²=1.63, df=2	0.03	χ²=13.76, df=6
Working	215 (74.7)	98 (79.7)	19 (67.9)			36 (70.6)			62 (72.1)				
Studying	29 (10.1)	7 (5.7)	4 (14.3)			11 (21.6)			7 (8.1)				
Unemployed/retired	44 (15.3)	18 (14.6)	5 (17.9)			4 (7.8)			17 (19.8)				
Do you have health insurance?				0.09	NA		<0.001	χ²=44.07, df=1		<0.001	χ²=28.21, df=1	<0.001	χ²=43.56, df=3
Yes	247 (85.8)	122 (99.2)	26 (92.9)			33 (64.7)			66 (76.7)				
No	41 (14.2)	1 (0.8)	2 (7.1)			18 (35.3)			20 (23.3)				

Diagnostic experiences

Across all groups, nearly all respondents reported receiving a professional endometriosis diagnosis, most often between ages 25 and 34 years (Table 2).

**Table 2:** Experiences With Diagnosis and Access to Endometriosis Care Among Survey Participants by Ethnicity and Country of Residence Chi-square or Fisher’s exact test was used to assess differences across the four ethnicity/country of residence groups. *Represents differences across the four ethnicity/country of residence groups. †Pairwise comparisons were conducted between each group and U.S. non-Hispanic participants (categorical variables). ‡χ² statistics are shown where χ² tests were used. For comparisons with sparse cell counts, Fisher’s exact test was applied; a χ² statistic is therefore not reported for those comparisons. P-values<0.05 considered statistically significant. Questions related to diagnostic pathway were answered by all respondents based on their symptom history and diagnostic experience, regardless of formal healthcare professional diagnosis status. U.S.: United States; χ² = chi-square; df: degrees of freedom; NA: not applicable

		U.S. residents				Non-U.S. residents							
Experiences	All (n=288)	Non-Hispanic (n=123)	Hispanic (n=28)	p-value†	Test statistic‡	Non-Hispanic (n=51)	p-value†	Test statistic‡	Hispanic (n=86)	p-value†	Test statistic‡	p-value*	Test statistic‡
Were you diagnosed with endometriosis by a healthcare professional?				0.61	NA		0.67	NA		0.40	NA	0.30	NA
Yes	279 (96.9)	118 (95.9)	26 (92.9)			50 (98.0)			85 (98.8)				
No	9 (3.1)	5 (4.1)	2 (7.1)			1 (2.0)			1 (1.2)				
At what age were you diagnosed with endometriosis?				0.83	χ²=0.36, df=2		0.78	χ²=0.51, df=2		0.97	χ²=0.06, df=2	0.99	χ²=1.00, df=6
Under 25	85 (29.5)	38 (30.9)	8 (28.6)			13 (25.5)			26 (30.2)				
25-34	129 (44.8)	54 (43.9)	14 (50.0)			24 (47.1)			37 (43.0)				
35+	74 (25.7)	31 (25.2)	6 (21.4)			14 (27.5)			23 (26.7)				
How long did it take to receive a diagnosis of endometriosis after your first symptoms?				0.45	NA		0.75	NA		0.52	χ²=2.27, df=3	0.54	NA
Less than 1 year	28 (9.7)	12 (9.8)	2 (7.1)			4 (7.8)			10 (11.6)				
1-2 years	16 (5.6)	6 (4.9)	1 (3.6)			2 (3.9)			7 (8.1)				
2-5 years	49 (17)	19 (15.4)	8 (28.6)			5 (9.8)			17 (19.8)				
> 5 years	195 (67.7)	86 (69.9)	17 (60.7)			40 (78.4)			52 (60.5)				
How many healthcare providers did you see before receiving a diagnosis?				0.44	χ²=2.72, df=3		0.06	χ²=7.37, df=3		0.59	χ²=1.91, df=3	0.07	χ²=15.84, df=9
1	25 (8.7)	10 (8.1)	1 (3.6)			4 (7.8)			10 (11.6)				
2-3	70 (24.3)	29 (23.6)	10 (35.7)			6 (11.8)			25 (29.1)				
4-5	65 (22.6)	31 (25.2)	8 (28.6)			8 (15.7)			18 (20.9)				
> 5	128 (44.4)	53 (43.1)	9 (32.1)			33 (64.7)			33 (38.4)				
Have you been evaluated by an endometriosis specialist?				0.26	χ²=1.27, df=1		0.57	χ²=0.33, df=1		0.002	χ²=9.22, df=1	0.02	χ²=9.71, df=3
Yes	193 (67.0)	92 (74.8)	18 (64.3)			36 (70.6)			47 (54.7)				
No	95 (33.0)	31 (25.2)	10 (35.7)			15 (29.4)			39 (45.3)				
Do you feel that you have adequate access to healthcare provider’s knowledge about endometriosis?				0.99	χ²=0.02, df=2		0.08	χ²=5.01, df=2		0.10	χ²=4.60, df=2	0.23	χ²=8.06, df=6
Yes	60 (20.8)	32 (26.0)	7 (25.0)			6 (11.8)			15 (17.4)				
No	200 (69.4)	83 (67.5)	19 (67.9)			39 (76.5)			59 (68.6)				
Unsure	28 (9.7)	8 (6.5)	2 (7.1)			6 (11.8)			12 (14.0)				
Do you need to travel out of state or to a different country to see an endometriosis specialist?				0.04	χ²=4.66, df=1		0.22	χ²=1.52, df=1		0.04	χ²=4.28, df=1	0.006	χ²=12.33, df=3
Yes, out of state/country	86 (29.9)	32 (26.0)	2 (7.1)			18 (35.3)			34 (39.5)				
No	202 (70.1)	91 (74.0)	26 (92.9)			33 (64.7)			52 (60.5)				
How far do you have to travel to see a specialist for endometriosis care?				0.01	χ²=10.60, df=3		0.24	χ²=4.18, df=3		<0.001	χ²=43.55, df=4	<0.001	χ²=67.96, df=12
Less than 10 miles	69 (24.0)	24 (19.5)	8 (28.6)			15 (29.4)			22 (25.6)				
10-25 miles	82 (28.5)	28 (22.8)	13 (46.4)			15 (29.4)			26 (30.2)				
26-50 miles	41 (14.2)	21 (17.1)	3 (10.7)			7 (13.7)			10 (11.6)				
50-100 miles	78 (27.1)	50 (40.7)	4 (14.3)			14 (27.5)			10 (11.6)				
> 100 miles	18 (6.3)	0	0			0			18 (20.9)				

Diagnostic delays were long, with the majority in each group reporting >five years from symptom onset to diagnosis. About one-third or more, 9 (32%) U.S. Hispanics to 33 (65%) non-U.S. non-Hispanics, saw five or more providers before receiving a diagnosis, indicating widespread diagnostic fragmentation. These patterns did not differ significantly between groups.

Specialist access differed meaningfully: only 47 (55%) non-U.S. Hispanic respondents had seen an endometriosis specialist, compared with 92 (75%) U.S. non-Hispanics (p = 0.002). Perceived access to knowledgeable clinicians was low across all groups, with fewer than one-third in any group reporting adequate access.

Travel and geographic burden

Travel burden showed the clearest disparities (Table 2). U.S. Hispanics were less likely to require out-of-state or international travel for specialist care, as shown by 2 (7%) U.S. Hispanics traveling out-of-state compared to 32 (26%) U.S. non-Hispanics (p=0.04). In contrast, 34 (40%) non-U.S. Hispanic participants required cross-border travel when compared to the U.S. non-Hispanic group; this represented the greatest difference in travel burden across all groups (p=0.04).

Distance traveled also differed significantly. Compared with U.S. non-Hispanics, U.S. Hispanic respondents were more likely to live within 25 miles of a specialist, 21 (75%) U.S. Hispanics to 52 (42%) U.S. non-Hispanics (p=0.01). In contrast, 18 (21%) non-U.S. Hispanics reported traveling more than 100 miles, whereas no U.S. non-Hispanic respondents traveled that far (p≤0.001). These findings highlight substantial geographic differences in reported access to specialized care, particularly for Hispanic respondents outside the U.S.

Barriers to accessing endometriosis care 

All comparison groups reported greater difficulty obtaining a referral to an endometriosis specialist than U.S. non-Hispanic participants, with the greatest burden observed among non-U.S. Hispanic respondents (Table 3). Referral challenges were especially pronounced among non-U.S. Hispanics, as seen by 68 (79%) non-U.S. Hispanics reporting difficulty obtaining a referral as compared to 49 (40%) U.S. non-Hispanics (p≤0.001). Financial barriers followed a similar pattern, affecting 60 (70%) non-U.S. Hispanics as compared to 65 (53%) U.S. non-Hispanic respondents (p=0.01). A high proportion of participants reported lacking health insurance, with the highest prevalence observed among U.S. Hispanics, of whom 17 (61%) reported being uninsured, compared to 53 (43%) U.S. non-Hispanics; however, this difference did not reach statistical significance. Long wait times were commonly reported across all groups, with 182 (63%) respondents overall indicating delays. Among the specific groups, 44 (51%) non-U.S. Hispanics reported long wait times compared to 81 (66%) U.S. non-Hispanics, a difference that was statistically significant (p=0.03).

**Table 3:** Barriers to Endometriosis Care Among Survey Participants by Ethnicity and Country of Residence Chi-square or Fisher’s exact test was used to assess differences across the four ethnicity/country of residence groups. *Represents differences across the four ethnicity/country of residence groups. †Pairwise comparisons were conducted between each group and U.S. non-Hispanic participants (categorical variables). ‡χ² statistics are shown, where χ² tests were used. For comparisons with sparse cell counts, Fisher’s exact test was applied; a χ² statistic is therefore not reported for those comparisons. P-values<0.05 considered statistically significant. U.S.: United States; χ² = chi-square; df: degrees of freedom; NA: not applicable

		U.S. residents				Non-U.S. residents							
Barriers to accessing care for endometriosis	All (n=288)	Non-Hispanic (n=123)	Hispanic (n=28)	p-value†	Test statistic‡	Non-Hispanic (n=51)	p-value†	Test statistic‡	Hispanic (n=86)	p-value†	Test statistic‡	p-value*	Test statistic‡
Difficulty obtaining a referral to a specialist for endometriosis				0.04	χ²=4.04, df=1		0.01	χ²=6.37, df=1		<0.001	χ²=31.61, df=1	<0.001	χ²=32.37, df=3
No	123 (42.7)	74 (60.2)	11 (39.3)			20 (39.2)			18 (20.9)				
Yes	165 (57.3)	49 (39.8)	17 (60.7)			31 (60.8)			68 (79.1)				
Financial constraints				0.15	χ²=2.08, df=1		0.06	χ²=3.67, df=1		0.01	χ²=6.03, df=1	0.04	χ²=7.95, df=3
No	109 (37.8)	58 (47.2)	9 (32.1)			16 (31.4)			26 (30.2)				
Yes	179 (62.2)	65 (52.8)	19 (67.9)			35 (68.6)			60 (69.8)				
Lack of insurance				0.09	χ²=2.85, df=1		0.34	χ²=0.91, df=1		0.41	χ²=0.67, df=1	0.14	χ²=5.42, df=3
No	158 (54.9)	70 (56.9)	11 (39.3)			33 (64.7)			44 (51.2)				
Yes	130 (45.1)	53 (43.1)	17 (60.7)			18 (35.3)			42 (48.8)				
Long wait times for appointments				0.84	χ²=0.04, df=1		0.26	χ²=1.25, df=1		0.03	χ²=4.54, df=1	0.03	χ²=8.80, df=3
No	106 (36.8)	42 (34.1)	9 (32.1)			13 (25.5)			42 (48.8)				
Yes	182 (63.2)	81 (65.9)	19 (67.9)			38 (74.5)			44 (51.2)				
Distance of healthcare providers				0.21	χ²=1.57, df=1		0.79	χ²=0.07, df=1		0.19	χ²=1.75, df=1	0.33	χ²=3.46, df=3
No	158 (54.9)	63 (51.2)	18 (64.3)			25 (49.0)			52 (60.5)				
Yes	130 (45.1)	60 (48.8)	10 (35.7)			26 (51.0)			34 (39.5)				
Lack of knowledgeable healthcare providers				0.13	NA		0.54	χ²=0.38, df=1		0.02	χ²=5.30, df=1	0.09	χ²=6.39, df=3
No	51 (17.7)	15 (12.2)	7 (25.0)			8 (15.7)			21 (24.4)				
Yes	237 (82.3)	108 (87.8)	21 (75.0)			43 (84.3)			65 (75.6)				
Other				0.74	NA		0.67	χ²=0.19, df=1		0.83	χ²=0.04, df=1	0.84	χ²=0.84, df=3
No	256 (88.9)	109 (88.6)	26 (92.9)			44 (86.3)			77 (89.5)				
Yes	32 (11.1)	14 (11.4)	2 (7.1)			7 (13.7)			9 (10.5)				

Distance to care was commonly identified as a barrier across all groups, though no significant differences were observed between groups (Table 3). Perceived lack of knowledgeable providers was reported at high rates across all populations. Although access was low across all groups, modest differences were observed, with non-U.S. Hispanic participants reporting significantly lower access compared with the reference group (Table 3). Overall, referral difficulties, financial constraints, distance-related concerns, and perceived limitations in provider expertise were frequently reported across groups.

## Discussion

This study describes the differences in perceived access to endometriosis-related care among survey respondents, with particularly pronounced barriers reported by Hispanic participants living outside the US. While previous research has examined broad racial and ethnic disparities in gynecologic and reproductive health, few studies have specifically examined how Hispanic women - in the U.S. or internationally - experience access to endometriosis care. By characterizing diagnostic experiences, referral pathways, travel burden, financial barriers, and perceived availability of knowledgeable clinicians, our findings help contextualize structural inequities identified in population-level surgical datasets. Previous studies have shown that Hispanic and other minority patients have reduced access to minimally invasive surgery and poorer perioperative outcomes. For example, Orlando et al. reported that Hispanic women were significantly less likely to receive minimally invasive procedures and experienced higher perioperative complication rates, while Westwood et al. described consistent patterns of minority patients facing reduced access to minimally invasive options and suboptimal surgical outcomes [2,9]. While our data cannot identify causal pathways, our respondents’ experiences are consistent with patterns observed in surgical outcome studies and may reflect upstream access barriers that co-occur with those disparities. Given the nonprobability sampling strategy, reliance on self-reported data, and lack of multivariable adjustment, these findings should be interpreted as exploratory and hypothesis-generating rather than definitive estimates of disparities in endometriosis care.

Across groups, diagnostic challenges were substantial: long delays, high rates of seeing more than five providers before diagnosis, and low perceived access to knowledgeable clinicians were common among all participants. However, several disparities emerged when comparing groups to U.S. non-Hispanic participants (the reference group). Non-U.S. Hispanic participants differed most significantly across multiple domains. They were substantially older, had lower educational attainment, and demonstrated the most pronounced barriers to care, including the lowest rates of specialist evaluation and the highest rates of referral difficulty and financial burden. These access patterns may in part reflect underlying differences in age, educational attainment, and employment status between groups, which were not adjusted for in this exploratory analysis. Travel-related challenges were also most significant in this group, with over one-third requiring cross-border travel and one-fifth traveling more than 100 miles compared with no U.S. non-Hispanic respondents reporting such extreme distances. These findings suggest substantial access barriers at the health system and geographic level for Hispanic patients living outside the US, which may demonstrate geographic maldistribution of gynecologic subspecialists, particularly in rural and resource-limited settings that lead to long travel distances, as described in previous work [14]. This increased travel burden may be an interrelated mechanism contributing to the low rates of specialist evaluation due to decreased accessibility and number of specialists in non-U.S. communities, but this present study was not designed to identify a specific causal mechanism. Differences in healthcare infrastructure, specialist availability, referral pathways, insurance systems, and language or cultural barriers may contribute to the disparities observed between groups and warrant further supply-side investigation to determine causality.

U.S. Hispanic participants demonstrated fewer differences from U.S. non-Hispanics, but important inequities remain. They differed significantly in age and racial distribution and reported higher referral difficulty. Notably, those who accessed specialist care tended to live closer to a specialist, raising the possibility that geographic access may not be the primary barrier for this group, and that referral pathways or care navigation may play a larger role. Although differences in financial barriers and insurance status were not consistently statistically significant, U.S. Hispanics demonstrated a clinically meaningful trend toward higher financial burden relative to the reference group, suggesting that cost-related challenges may still play an important role.

Participants frequently viewed long diagnostic delays, difficulty securing referrals, and limited access to specialists as indications of inequitable or fragmented systems of care rather than isolated obstacles. These findings suggest that referral difficulties, particularly those reported by both U.S. and non-U.S. Hispanic participants, are associated with lower reported access to specialized care and may help contextualize differences in satisfaction and care delivery.

This is consistent with Zaritsky et al., who found lower referral rates among non-White women with endometriosis, although their study focused primarily on Black women [15]. Additionally, the geographic challenges identified in this study may partially contextualize the higher rates of perioperative complications among Hispanic patients reported by Orlando et al [9]. Similar barriers related to geography, cost, insurance, and provider communication have been documented in reproductive healthcare for Latina women, even outside the context of endometriosis [16]. More broadly, race, ethnicity, socioeconomic status, and geographic location contribute to disparities in care quality and outcomes for women with endometriosis, potentially underlying the geographic and financial barriers that could lead to feelings of stigmatization [2]. Differences in referral pathways, insurance coverage, and specialist access have also been highlighted as factors associated with delays and limiting access to care across all populations, not just Hispanics [3]. Finally, younger women and ethnic minorities report lower levels of patient-centered care and involvement in decision-making, which may further explain decreased access to specialized care [4]. Language discordance between patients and clinicians has been shown to negatively impact patient-centered communication, satisfaction, and shared decision-making, and may partially explain these differences [17]. Although some barriers overlap with those experienced by other minority groups, our study emphasizes the unique combination of geographic, financial, and referral challenges perceived by Hispanic women with endometriosis internationally. These patterns are consistent with broader effects of structural racism on healthcare access and quality [18].

There are a few important limitations to consider. The cross-sectional convenience sample limits generalizability, and findings should be interpreted as perceptions rather than population-level estimates. The adapted survey instrument has not yet been formally validated, and Hispanic respondents were not categorized by subgroup (e.g., Mexican, Puerto Rican, Colombian). Online recruitment may have excluded participants with limited internet access or lower health literacy, potentially underrepresenting more vulnerable populations. Analyses were unadjusted and exploratory, and therefore did not account for potential confounding variables such as socioeconomic status, insurance coverage, language and cultural barriers, healthcare infrastructure, or disease severity. These variables that differ between ethnic and geographic groups require further research to disentangle these complex relationships.

Future research should prioritize strategies to improve culturally responsive endometriosis care and reduce referral, financial, and geographic barriers to specialist access. The survey instrument used in this study may serve as a foundation for future investigations across diverse healthcare settings. Interventions such as patient navigation programs, telehealth expansion, and bilingual community-based support warrant further evaluation for their potential to improve access and patient experience. Additionally, longitudinal studies are needed to assess how access to specialized care influences clinical outcomes, patient satisfaction, and quality of life over time. More granular analyses of Hispanic subgroups, including language preference, immigration status, and socioeconomic context, may further elucidate heterogeneity in barriers and care experiences.

## Conclusions

In this international survey, Hispanic respondents - particularly those living outside the US - reported greater referral challenges, more financial constraints, and more geographic barriers to accessing endometriosis care compared with U.S. non-Hispanic respondents. These experiences included a lower likelihood of specialist evaluation and substantial travel burden, underscoring meaningful structural barriers to endometriosis care. Across all groups, limited perceived access to knowledgeable clinicians highlights a broader shortage of endometriosis expertise. Together, these patient-reported perspectives highlight potential targets for future interventions, including referral pathways, specialist availability, and financial and geographic barriers, to support more equitable and patient-centered endometriosis care.

## Appendices

Endometriosis access to care project

Survey Flow

Block: Language Preference (1 question)

Branch: New Branch

If

If Do you prefer to answer this questionnaire in Spanish or English? ¿En qué idioma prefiere complet... English/Inglés Is Selected

Block: Introduction to Project - English (1 question)

Block: Informed Consent - English (1 question)

Block: General Questions - English (9 questions)

Block: Endometriosis Diagnosis and Treatment - English (3 questions)

Block: Access to Care - English (11 questions)

Block: Access to Treatment - English (5 questions)

Block: Perception of Care - English (8 questions)

Standard: Conclusion - English (1 question)

Branch: New Branch

If

If Do you prefer to answer this questionnaire in Spanish or English? ¿En qué idioma prefiere complet... Español/Spanish Is Selected

Standard: Introduction to Project - Spanish (1 question)

Standard: Informed Consent - Spanish (1 question)

Standard: General Questions - Spanish (9 questions)

Standard: Endometriosis Diagnosis and Treatment - Spanish (3 questions)

Standard: Access to Care - Spanish (11 questions)

Standard: Access to Treatment - Spanish (5 questions)

Standard: Perception of Care - Spanish (8 questions)

Standard: Conclusion - Spanish (1 question)

Page Break

Start of Block: Language Preference

Do you prefer to answer this questionnaire in Spanish or English? ¿En qué idioma prefiere completar la encuesta?

o Español/Spanish (1)

o English/Inglés (2)

End of Block: Language Preference

Start of Block: Introduction to Project - English

Thank you for volunteering to participate in this research study. The completion of this survey will take approximately 5 minutes. 

STUDY TITLE: Evaluation of patient access to endometriosis care

IRB STUDY #: 007633 PRINCIPAL INVESTIGATOR: Dr. Diana Encalada Soto, MD Department of Obstetrics and Gynecology STUDENT INVESTIGATORS: Eliana Burgos, MS2, USF Health Morsani College of Medicine PRIMARY OBJECTIVE: Purpose of the survey: The purpose of this survey is to gather information on the challenges and experiences of patients with endometriosis in accessing care. Your input is crucial in helping us understand and improve healthcare services for endometriosis patients. Importance of participant input: Your participation will contribute to valuable research aimed at enhancing care and support for those affected by endometriosis.

End of Block: Introduction to Project - English

Start of Block: Informed Consent - English

Informed Consent to Participate in Research Involving Minimal Risk and Authorization to Collect, Use and Share Your Health Information Information to Consider Before Taking Part in this Research Study Title: Evaluation of patient access to endometriosis care Study # 007633 Overview: You are being asked to take part in a research study. The information in this document should help you to decide if you would like to participate. The sections in this Overview provide the basic information about the study. More detailed information is provided in the remainder of the document. Study Staff: This study is being led by Dr. Encalada Soto who is a minimally invasively gynecological surgeon at the University of South Florida]. This person is called the Principal Investigator. USF medical student Eliana Burgos may act on behalf of the Principal Investigator. Study Details: The purpose of this survey is to gather information on the challenges and experiences of patients with endometriosis in accessing care. Your input is crucial in helping us understand and improve healthcare services for endometriosis patients. Subjects: You are being asked to take part because you have a diagnosis of endometriosis. We want to understand your access to endometriosis care. Voluntary Participation: Your participation is voluntary. You do not have to participate and may stop your participation at any time. There will be no penalties or loss of benefits or opportunities if you do not participate or decide to stop once you start. Benefits, Compensation, and Risk: We do not know if you will receive any benefit from your participation. There is no cost to participate. You will not be compensated for your participation. This research is considered minimal risk. Minimal risk means that study risks are the same as the risks you face in daily life. Your participation will allow health providers to better understand the complex effects endometriosis can have on your quality of life and overall health. Confidentiality: Even if we publish the findings from this study, we will keep your study information private and confidential. Anyone with the authority to look at your records must keep them confidential. The study data will be stored for up to five years after study completion by the study team. Privacy and Confidentiality: It is possible, although unlikely, that unauthorized individuals could gain access to your responses because you are responding online. Confidentiality will be maintained to the degree permitted by the technology used. No guarantees can be made regarding the interception of data sent via the Internet. However, your participation in this online survey involves risks similar to a person’s everyday use of the Internet. If you complete and submit an anonymous survey and later request your data be withdrawn, this may or may not be possible as the researcher may be unable to extract anonymous data from the database. Contact Information If you have any questions, concerns or complaints about this study, email Eliana Burgos, elianaburgos@usf.edu. If you have questions about your rights, complaints, or issues as a person taking part in this study, call the USF IRB at (813) 974-5638 or contact the IRB by email at RSCH-IRB@usf.edu. We may publish what we learn from this study. If we do, we will not let anyone know your name. We will not publish anything else that would let people know who you are. You can print a copy of this consent form for your records. I freely give my consent to take part in this study. I understand that by proceeding with this survey, I am agreeing to take part in research and I am 18 years of age or older. Authorization to Use and Disclose Protected Health Information (HIPAA Language) The federal privacy regulations of the Health Insurance Portability & Accountability Act (HIPAA) protect your identifiable health information. By providing your authorization, you are permitting the University of South Florida to use your health information for research purposes. You are also allowing us to share your health information with individuals or organizations other than USF who are also involved in the research and listed below. In addition, the following groups of people may also be able to see your health information and may use that information to conduct this research: The medical staff that takes care of you and those who are part of this research study Anyone listed above may use consultants in this research study, and may share your information with them. If you have questions about who they are, you should ask the study team. Individuals who receive your health information for this research study may not be required by the HIPAA Privacy Rule to protect it and may share your information with others without your permission. They can only do so if permitted by law. If your information is shared, it may no longer be protected by the HIPAA Privacy Rule. By providing your authorization, you are giving your permission to use and/or share your health information as described in this document. As part of this research, USF may collect, use, and share the following information Your research record All of your past, current or future medical and other health records held by USF, other health care providers or any other site affiliated with this study as they relate to this research project. This may include, but is not limited to records related to HIV/AIDs, mental health, substance abuse, and/or genetic information. You can refuse to provide your authorization. If you do not provide your authorization, you will not be able to take part in this research study. However, your care outside of this study and benefits will not change. Your authorization to use your health information will not expire unless you revoke (withdraw) it in writing. You can revoke your authorization at any time by sending a letter clearly stating that you wish to withdraw your authorization to use your health information in the research. If you revoke your permission: You will no longer be a subject in this research study; we will stop collecting new information about you; We will use the information collected prior to the revocation of your authorization. This information may already have been used or shared with others, or we may need it to complete and protect the validity of the research; and Staff may need to follow-up with you if there is a medical reason to do so. To revoke your authorization, please write an email to: Eliana Burgos For IRB Study # 007633 elianaburgos@usf.edu While we are conducting the research study, we cannot let you see or copy the research information we have about you. After the research is completed, you have a right to see the information about you, as allowed by USF policies. By continuing the survey and clicking the arrow, you are consenting to participation in this study. 

End of Block: Informed Consent - English

Start of Block: General Questions - English 

What is your age range?

o 18-25 (1)

o 26-35 (2)

o 36-45 (3)

o 46-55 (4)

o 56-65 (5)

o 66+ (6)

Race - select all that apply

▢ White (1)

▢ Black or African American (2)

▢ American Indian or Alaska Native (3)

▢ Asian (4)

▢ Native Hawaiian or Pacific Islander (5)

▢ Other (6)

Page Break

Ethnicity

o Not Hispanic or Latino (1)

o Hispanic or Latino (2)

Country of Residence

o United States (4)

o Other (Specify Country) (2) __________________________________________________

Display this question:

If Country of Residence = United States

In which state do you currently reside?

o Alabama (1)

o Alaska (2)

o Arizona (3)

o Arkansas (4)

o California (5)

o Colorado (6)

o Connecticut (7)

o Delaware (8)

o District of Columbia (9)

o Florida (10)

o Georgia (11)

o Hawaii (12)

o Idaho (13)

o Illinois (14)

o Indiana (15)

o Iowa (16)

o Kansas (17)

o Kentucky (18)

o Louisiana (19)

o Maine (20)

o Maryland (21)

o Massachusetts (22)

o Michigan (23)

o Minnesota (24)

o Mississippi (25)

o Missouri (26)

o Montana (27)

o Nebraska (28)

o Nevada (29)

o New Hampshire (30)

o New Jersey (31)

o New Mexico (32)

o New York (33)

o North Carolina (34)

o North Dakota (35)

o Ohio (36)

o Oklahoma (37)

o Oregon (38)

o Pennsylvania (39)

o Puerto Rico (40)

o Rhode Island (41)

o South Carolina (42)

o South Dakota (43)

o Tennessee (44)

o Texas (45)

o Utah (46)

o Vermont (47)

o Virginia (48)

o Washington (49)

o West Virginia (50)

o Wisconsin (51)

o Wyoming (52)

o I do not reside in the United States (53)

What is the highest level of education you have completed?

o None (1)

o Elementary School (4)

o Middle School (5)

o High School (6)

o Community College/Technical School (7)

o Bachelor’s Degree (8)

o Graduate School (Master’s, Doctoral, or professional degree) (9)

o Other, specify: (10) __________________________________________________

Employment Status

o Working (1)

o Studying (2)

o Unemployed (3)

o Retired (4)

Were you diagnosed with endometriosis by a healthcare professional?

o No (1)

o Yes (2)

Display this question:

If Were you diagnosed with endometriosis by a healthcare professional? = Yes

Since you responded "Yes" to being diagnosed with endometriosis by a healthcare professional, please specify what healthcare professional.

o Primary Care Provider or General Practitioner (1)

o OBGYN (2)

o Endometriosis Specialist (3)

o Other (Please Specify) (4) __________________________________________________

End of Block: General Questions - English 

Start of Block: Endometriosis Diagnosis and Treatment - English

How long did it take to receive a diagnosis of endometriosis after your first symptoms?

o Less than 6 months (1)

o 6 months to 1 year (2)

o 1-2 years (3)

o 2-5 years (4)

o More than 5 years (5)

How many healthcare providers did you see before receiving a diagnosis?

o 1 (1)

o 2-3 (2)

o 4-5 (3)

o More than 5 (4)

At what age were you diagnosed with endometriosis?

o Under 18 (1)

o 18 - 24 (2)

o 25 - 34 (3)

o 35 - 44 (4)

o 45 - 54 (5)

o 55-65 (6)

End of Block: Endometriosis Diagnosis and Treatment - English

Start of Block: Access to Care - English

Do you feel that you have adequate access to healthcare providers knowledgeable about endometriosis?

o Yes (1)

o No (2)

o Unsure (3)

Have you been evaluated by an endometriosis specialist (i.e., a medical professional with advanced training and expertise in diagnosing and treating endometriosis?

o Yes (1)

o No (2)

Display this question:

If Have you been evaluated by an endometriosis specialist (i.e., a medical professional with advanced... = Yes

How did you find the endometriosis specialist?

o Advertisement (1)

o Social Media (i.e., Facebook, Instagram) (2)

o Word of mouth (3)

o Referral by another physician (i.e. regular OBGYN, primary care provider, etc). (4)

Display this question:

If Have you been evaluated by an endometriosis specialist (i.e., a medical professional with advanced... = Yes

How long was your wait time to be seen in clinic by the endometriosis specialist?

o Less than 1 month (1)

o 1-2 months (2)

o 3-6 months (3)

o 7-12 months (4)

o Greater than 12 months (5)

How far do you have to travel to see a specialist for endometriosis care?

o Less than 10 miles (1)

o 10-25 miles (2)

o 26-50 miles (3)

o More than 50 miles (4)

Do you need to travel out of state or to a different country to see an endometriosis specialist?

o Yes, out of state (1)

o Yes, to a different country (2)

o No (3)

Have you ever had difficulty obtaining a referral to an specialist for endometriosis?

o Yes (1)

o No (2)

Have you experienced any of the following barriers to accessing care for endometriosis? Select all that apply.

▢ Financial constraints (1)

▢ Lack of insurance coverage (2)

▢ Long wait times for appointments (3)

▢ Distance of healthcare providers (4)

▢ Lack of knowledge healthcare providers (5)

▢ Other (Please Specify) (6) __________________________________________________

Do you have health insurance?

o Yes (1)

o No (2)

Display this question:

If Do you have health insurance? = Yes

What type of insurance do you have?

o Private (1)

o Medicaid (2)

o Medicare (3)

o Other (Please Specify) (4) __________________________________________________

Have you ever been denied insurance coverage for any endometriosis-related treatment?

o Yes (1)

o No (2)

End of Block: Access to Care - English

Start of Block: Access to Treatment - English

What treatments have you received for endometriosis? Select all that apply.

▢ Medications (e.g., pain relievers, hormonal therapy) (1)

▢ Surgery (2)

▢ Physical therapy (3)

▢ Complementary therapies (e.g., acupuncture, dietary changes) (4)

▢ Other (please specify) (5) __________________________________________________

Display this question:

If What treatments have you received for endometriosis? Select all that apply. = Surgery

How many surgeries have you undergone for endometriosis?

o 1 (1)

o 2 (2)

o 3 (3)

o 4 (4)

o 5 (5)

o 6 or more (6)

How satisfied are you with the effectiveness of your current treatment plan?

o Very satisfied (1)

o Satisfied (2)

o Neutral (3)

o Dissatisfied (4)

o Very dissatisfied (5)

How involved do you feel in making decisions about your endometriosis treatment plan?

o Very involved (1)

o Somewhat involved (2)

o Not very involved (3)

o Not involved at all (4)

Do you have access to support groups or counseling for endometriosis?

o Yes (1)

o No (2)

End of Block: Access to Treatment - English

Start of Block: Perception of Care - English

Before meeting with an endometriosis specialist: How would you rate your overall satisfaction with the care you received for your endometriosis?

o Very satisfied (1)

o Satisfied (2)

o Neural (3)

o Dissatisfied (4)

o Very dissatisfied (5)

After meeting with an endometriosis specialist: How would you rate your overall satisfaction with the care you received for your endometriosis?

o Very satisfied (1)

o Satisfied (2)

o Neutral (3)

o Dissatisfied (4)

o Very dissatisfied (5)

Have you had access to complementary care for your endometriosis diagnosis?

▢ No access to complementary care (1)

▢ Pelvic floor physical therapy (4)

▢ Nutritionist (5)

▢ Pain management specialist (6)

▢ Counseling/psychotherapy (7)

▢ Other (please specify): (8) __________________________________________________

Do you feel your healthcare providers listen to your concerns and symptoms related to endometriosis?

o Always (1)

o Most of the time (2)

o Sometimes (3)

o Rarely (4)

o Never (5)

Have you ever felt dismissed or not taken seriously by a healthcare provider when discussing your endometriosis symptoms?

o Yes (1)

o No (2)

Have you ever experienced stigma or discrimination when seeking care for endometriosis?

o Yes (1)

o No (2)

Do you feel there are enough resources available to you for managing endometriosis?

o Yes (1)

o No (2)

o Unsure (3)

Is there anything else you would like to share about your experience with accessing care for endometriosis?

o Yes (Please Specify) (1) __________________________________________________

o No (2)

End of Block: Perception of Care - English

Start of Block: Conclusion - English

Thank you for taking the time to complete this survey. Your input is invaluable in helping us improve access to care for endometriosis patients. If you have any questions or additional comments, please feel free to contact our research team: Elianaburgos@usf.edu Eliana Burgos, MS2

End of Block: Conclusion - English

Start of Block: Introduction to Project - Spanish

Gracias por ofrecerte como voluntario para participar en este estudio de investigación. Completar esta encuesta tomará aproximadamente 5 minutos. TÍTULO DEL ESTUDIO: Evaluación del acceso de los pacientes a la atención de la endometriosis N.º DE ESTUDIO IRB: 007633 INVESTIGADOR PRINCIPAL: Dra. Diana Encalada Soto, MD Departamento de Obstetricia y Ginecología INVESTIGADORES ESTUDIANTES: Eliana Burgos, MS2, USF Health Morsani College of Medicine OBJETIVO PRINCIPAL: Propósito de la encuesta: El propósito de esta encuesta es recopilar información sobre los desafíos y experiencias de los pacientes con endometriosis al acceder a la atención médica. Tu opinión es crucial para ayudarnos a comprender y mejorar los servicios de atención médica para los pacientes con endometriosis. Importancia de la participación: Tu participación contribuirá a una valiosa investigación destinada a mejorar la atención y el apoyo para quienes se ven afectados por la endometriosis.

End of Block: Introduction to Project - Spanish

Start of Block: Informed Consent - Spanish

Consentimiento informado para participar en investigación de riesgo y autorización para recolectar, usar y compartir su información médica Información para tener en cuenta antes de participar en este estudio de investigación Título: Evaluación del acceso de los pacientes de la endometriosis Estudio N° 007633 Resumen: Se le ha solicitado que participe en un estudio de investigación. La información de este documento le ayudará a decidir si desea participar. Las secciones de este Resumen le proporcionarán la información básica acerca del estudio. Se proporciona información más detallada en el resto del documento. Personal del estudio: Este estudio está dirigido por Dra. Encalada Soto que es cirujana ginecológica de cirugía mínimamente invasiva en la Universidad del Sur de Florida. A esta persona se la conoce como Investigador Principal (PI, por sus siglas en inglés). La estudiants de medicina de USF Eliana Burgos pueden actuar en nombre del Investigador Principal. Detalles del estudio: El propósito de esta encuesta es recopilar información sobre los desafíos y experiencias de los pacientes con endometriosis al acceder a la atención médica. Su participación es crucial para ayudarnos a comprender y mejorar los servicios de atención médica para los pacientes con endometriosis. Sujetos: Se le está pidiendo que participe porque tiene un diagnóstico de endometriosis. Queremos entender su acceso a la atención para la endometriosis. Participación voluntaria: Su participación es voluntaria. Usted no tiene que participar y puede detener su participación en cualquier momento. No habrá multas ni pérdida de beneficios u oportunidades si usted no participa o decide dejar de hacerlo una vez que comience. Beneficios, compensación y riesgo: Nosotros no sabemos si usted recibirá algún beneficio por su participación. La participación es gratuita. No se le compensará por su participación. Esta investigación se considera de riesgo mínimo. Riesgo mínimo significa que los riesgos del estudio son los mismos que los riesgos a los que se enfrenta en la vida diaria. Su participación les permitirá a los proveedores de salud tener una mejor comprensión sobre los efectos complejos que la endometriosis puede tener en su calidad de vida y salud en general. Confidencialidad: Incluso si publicamos los resultados de este estudio, mantendremos la información de su estudio de forma privada y confidencial. Cualquier persona con autoridad para ver sus registros debe mantener la confidencialidad de éstos. Privacidad y confidencialidad: Es posible, aunque poco probable, que personas no autorizadas puedan obtener acceso a sus respuestas porque usted está respondiendo en línea. La confidencialidad se mantendrá en la medida en que lo permita la tecnología utilizada. No se puede garantizar la interceptación de los datos enviados a través de Internet. Sin embargo, su participación en esta encuesta en línea implica riesgos similares al uso diario de Internet por parte de una persona. Si completa y envía una encuesta anónima y luego solicita que se retiren sus datos, esto puede o no ser posible, ya que es posible que el investigador no pueda extraer datos anónimos de la base de datos. Información de contacto Si usted tiene alguna pregunta, inquietud o queja sobre este estudio, envíe un correo electrónico a Eliana Burgos, elianaburgos@usf.edu. Si tiene preguntas sobre sus derechos, quejas o problemas como persona que participa en este estudio, llame al IRB de USF al (813) 974-5638 o comuníquese con el IRB por correo electrónico a RSCH-IRB@usf.edu. Es posible que nosotros publiquemos lo que aprendamos de este estudio. Si lo hacemos, no dejaremos que nadie sepa su nombre. Nosotros no publicaremos nada más que le permita a la gente saber quién es usted. Puede imprimir una copia de este formulario de consentimiento para sus registros. Doy mi consentimiento libre para participar en este estudio. Entiendo que, al proceder con esta encuesta, estoy aceptando participar en la investigación y que tengo 18 años de edad o más. Autorización para usar y divulgar información médica protegida (lenguaje de HIPAA) Las regulaciones federales de privacidad de la Ley de Portabilidad y Responsabilidad de Seguros Médicos (HIPAA, por sus siglas en inglés) protegen su información de salud identificable. Al proporcionar su autorización, usted está permitiendo que la Universidad del Sur de Florida utilice su información de salud con fines de investigación. También nos permite compartir su información de salud con personas u organizaciones que no sean USF que también estén involucradas en la investigación y que se enumeran a continuación. Además, los siguientes grupos de personas también pueden ver su información de salud y pueden usar esa información para llevar a cabo esta investigación: • El personal médico que lo atiende y las personas que forman parte de este estudio de investigación Cualquier persona mencionada anteriormente puede utilizar consultores en este estudio de investigación y puede compartir su información con ellos. Si tiene preguntas sobre quiénes son, debe preguntar al equipo del estudio. Es posible que las personas que reciben su información de salud para este estudio de investigación no estén obligadas por la Regla de Privacidad de HIPAA a protegerla y pueden compartir su información con otros sin su permiso. Solo pueden hacerlo si la ley lo permite. Si se comparte su información, es posible que ya no esté protegida por la Regla de privacidad de HIPAA. Al proporcionar su autorización, usted está dando su permiso para usar y/o compartir su información de salud como se describe en este documento. Como parte de esta investigación, USF puede recopilar, usar y compartir la siguiente información • Su historial de investigación • Todos sus registros médicos y otros registros de salud pasados, actuales o futuros en poder de la USF, otros proveedores de atención médica o cualquier otro sitio afiliado a este estudio en relación con este proyecto de investigación. Esto puede incluir, entre otros, registros relacionados con el VIH/SIDA, la salud mental, el abuso de sustancias y/o la información genética. Usted puede negarse a proporcionar su autorización. Si no proporciona su autorización, no podrá participar en este estudio de investigación. Sin embargo, su atención fuera de este estudio y sus beneficios no cambiarán. Su autorización para usar su información de salud no expirará a menos que la revoque (retire) por escrito. Usted puede revocar su autorización en cualquier momento enviando una carta indicando claramente que desea retirar su autorización para usar su información de salud en la investigación. Si revoca su permiso: • Usted ya no será un sujeto en este estudio de investigación;• Nosotros dejaremos de recopilar nueva información sobre usted;• Utilizaremos la información recopilada antes de la revocación de su autorización. Es posible que esta información ya se haya utilizado o compartido con otros, o que la necesitemos para completar y proteger la validez de la investigación; y • Es posible que el personal deba hacer un seguimiento con usted si existe una razón médica para hacerlo. Para revocar su autorización, por favor escriba un correo electrónico a: Eliana Burgos elianaburgos@usf.edu Mientras llevamos a cabo el estudio de investigación, no podemos permitirle ver ni copiar la información de investigación que tenemos sobre usted. Una vez completada la investigación, usted tiene derecho a ver la información sobre usted, según lo permitan las políticas de la USF. Al continuar con la encuesta y hacer clic en la flecha, usted está dando su consentimiento para participar en este estudio.

End of Block: Informed Consent - Spanish

Start of Block: General Questions - Spanish

¿Cuántos años tiene?

o 18 -25 (1)

o 26-35 (4)

o 36-45 (5)

o 46-55 (6)

o 56-65 (7)

o 66+ (8)

Raza - Seleccione todas las que correspondan

▢ Blanco (1)

▢ Negro o Afroamericano (4)

▢ Asiático (5)

▢ Nativo de Hawái u otra isla del Pacífico (6)

▢ Indígena Americano o Nativo de Alaska (7)

▢ Otro (especifique) (8)

Etnicidad

o Hispano o Latino (1)

o No Hispano o Latino (2)

País de residencia

o Estados Unidos. (4)

o Otro. Especifique su país. (2) __________________________________________________

Display this question:

If País de residencia = Estados Unidos.

Especifique su estado

o Alabama (1)

o Alaska (2)

o Arizona (3)

o Arkansas (4)

o California (5)

o Colorado (6)

o Connecticut (7)

o Delaware (8)

o District of Columbia (9)

o Florida (10)

o Georgia (11)

o Hawaii (12)

o Idaho (13)

o Illinois (14)

o Indiana (15)

o Iowa (16)

o Kansas (17)

o Kentucky (18)

o Louisiana (19)

o Maine (20)

o Maryland (21)

o Massachusetts (22)

o Michigan (23)

o Minnesota (24)

o Mississippi (25)

o Missouri (26)

o Montana (27)

o Nebraska (28)

o Nevada (29)

o New Hampshire (30)

o New Jersey (31)

o New Mexico (32)

o New York (33)

o North Carolina (34)

o North Dakota (35)

o Ohio (36)

o Oklahoma (37)

o Oregon (38)

o Pennsylvania (39)

o Puerto Rico (40)

o Rhode Island (41)

o South Carolina (42)

o South Dakota (43)

o Tennessee (44)

o Texas (45)

o Utah (46)

o Vermont (47)

o Virginia (48)

o Washington (49)

o West Virginia (50)

o Wisconsin (51)

o Wyoming (52)

o I do not reside in the United States (53)

¿Cuál es el nivel más alto de educación que ha completado?

o Ninguno (1)

o Escuela primaria (4)

o Escuela secundaria (5)

o Preparatoria/Bachillerato (6)

o Universidad comunitaria/Escuela técnica (7)

o Licenciatura (8)

o Posgrado (Maestría, Doctorado o título profesional) (9)

o Otro, especifique: (10) __________________________________________________

Estado de empleo

o Trabajando (1)

o Estudiando (2)

o Desempleado (3)

o Jubilado (4)

¿Fue diagnosticado por un profesional de la salud?

o No (1)

o Sí (2)

Display this question:

If ¿Fue diagnosticado por un profesional de la salud? = No

Si respondió Sí, ¿qué tipo de profesional de la salud?:

o Médico de atención primaria o médico general (1)

o Ginecólogo (2)

o Especialista en endometriosis (3)

o Otro (especifique) (4) __________________________________________________

End of Block: General Questions - Spanish

Start of Block: Endometriosis Diagnosis and Treatment - Spanish

¿Cuánto tiempo tardó en recibir un diagnóstico de endometriosis después de sus primeros síntomas?

o Menos de 6 meses (1)

o De 6 meses a 1 año (2)

o De 1 a 2 años (3)

o De 2 a 5 años (4)

o Más de 5 años (5)

¿Cuántos profesionales de la salud visitó antes de recibir un diagnóstico?

o 1 (1)

o 2-3 (2)

o 4-5 (3)

o Más de 5 (4)

¿A qué edad le diagnosticaron con endometriosis?

o Menos de 18 años (1)

o 18-24 años (2)

o 25-34 años (3)

o 35-44 años (4)

o 45-54 años (5)

End of Block: Endometriosis Diagnosis and Treatment - Spanish

Start of Block: Access to Care - Spanish

¿Siente que tiene acceso adecuado a proveedores de atención médica con conocimientos sobre endometriosis?

o Sí (1)

o No (2)

o No estoy seguro (3)

¿Ha sido evaluado por un especialista en endometriosis (un profesional médico con formación avanzada en el diagnóstico y tratamiento de la endometriosis)?

o Sí (1)

o No (2)

Display this question:

If ¿Ha sido evaluado por un especialista en endometriosis (un profesional médico con formación avanz... = Sí

¿Cómo encontró al especialista en endometriosis?

o Publicidad (1)

o Redes sociales (grupo de Facebook, Instagram) (2)

o Recomendación de otros pacientes (3)

o Referido por otro médico (ginecólogo habitual, médico de atención primaria u otro proveedor) (4)

Display this question:

If ¿Ha sido evaluado por un especialista en endometriosis (un profesional médico con formación avanz... = Sí

¿Cuánto tiempo esperó para ser atendido en la clínica por el especialista en endometriosis?

o Menos de 1 mes (1)

o 1-2 meses (2)

o 3-6 meses (3)

o 7-12 meses (4)

o Más de 12 meses (5)

¿Cuánto tiene que viajar para ver a un especialista en endometriosis?

o Menos de 10 millas (1)

o 10-25 millas (2)

o 26-50 millas (3)

o 51-100 millas (4)

o Más de 100 millas (5)

¿Necesita viajar fuera de su estado o a otro pais para ver a un especialista en endometriosis?

o Sí, fuera del estado (1)

o Sí, fuera del pais (2)

o No (3)

¿Ha tenido alguna vez dificultades para obtener una referencia a un especialista en endometriosis?

o Sí (1)

o No (2)

¿Ha experimentado alguna de las siguientes barreras para acceder a la atención por endometriosis? (Seleccione todas las que correspondan)

▢ Restricciones financieras (1)

▢ Falta de cobertura de seguro médico (2)

▢ Largos tiempos de espera para las citas (3)

▢ Distancia a los proveedores de atención médica (4)

▢ Falta de proveedores de atención médica con conocimientos (5)

▢ Otro (especifique) (6) __________________________________________________

¿Tiene seguro médico?

o Sí (1)

o No (2)

Display this question:

If ¿Tiene seguro médico? = Sí

Si tiene seguro médico, ¿qué tipo de seguro tiene?

o Seguro privado (1)

o Medicaid (2)

o Medicare (3)

o Otro (especifique) (4) __________________________________________________

¿Le han negado alguna vez la cobertura del seguro para algún tratamiento relacionado con la endometriosis?

o Sí (1)

o No (2)

End of Block: Access to Care - Spanish

Start of Block: Access to Treatment - Spanish

¿Qué tratamientos ha recibido para la endometriosis? (Seleccione todos los que correspondan)

▢ Medicamentos (por ejemplo, analgésicos, terapia hormonal) (1)

▢ Cirugía (2)

▢ Fisioterapia (3)

▢ Terapias complementarias (por ejemplo, acupuntura, cambios en la dieta) (4)

▢ Otro (especifique)(5) __________________________________________________

Display this question:

If ¿Qué tratamientos ha recibido para la endometriosis? (Seleccione todos los que correspondan) = Cirugía

¿Cuántas cirugías ha tenido para la endometriosis?

o 1 (1)

o 2 (2)

o 3 (3)

o 4 (4)

o 5 (5)

o 6 or más (6)

¿Qué tan satisfecho está con la efectividad de su plan de tratamiento actual?

o Muy satisfecho (1)

o Satisfecho (2)

o Neutral (3)

o Insatisfecho (4)

o Muy insatisfecho (5)

¿Qué tan involucrado se siente en la toma de decisiones sobre su plan de tratamiento para la endometriosis?

o Muy involucrado (1)

o Algo involucrado (2)

o Poco involucrado (3)

o No involucrado en absoluto (4)

¿Tiene acceso a grupos de apoyo o asesoramiento para la endometriosis?

o Sí (1)

o No (2)

End of Block: Access to Treatment - Spanish

Start of Block: Perception of Care - Spanish

Antes de consultar con un especialista en endometriosis: ¿Cómo calificaría su satisfacción general con la atención que recibe para la endometriosis?

o Muy satisfecho (1)

o Satisfecho (2)

o Neutral (3)

o Insatisfecho (4)

o Muy insatisfecho (5)

Después de consultar con un especialista en endometriosis: ¿Cómo calificaría su satisfacción general con la atención que recibió para su endometriosis?

o Muy satisfecho/a (1)

o Satisfecho/a (2)

o Neutral (3)

o Insatisfecho/a (4)

o Muy insatisfecho/a (5)

¿Ha tenido acceso a cuidados complementarios para su diagnóstico de endometriosis?

▢ No he tenido acceso a cuidados complementarios (1)

▢ Fisioterapia de piso pélvico (4)

▢ Nutricionista (5)

▢ Especialista en manejo del dolor (6)

▢ Consejería/Psicoterapia (7)

▢ Otro (especifique): (8) __________________________________________________

¿Cree que sus proveedores de atención médica escuchan sus preocupaciones y síntomas relacionados con la endometriosis?

o Siempre (1)

o La mayoría de las veces (2)

o A veces (3)

o Rara vez (4)

o Nunca (5)

¿Alguna vez se ha sentido ignorado o no tomado en serio por un proveedor de atención médica al hablar de sus síntomas de endometriosis?

o Sí (1)

o No (2)

¿Ha experimentado alguna vez estigma o discriminación al buscar atención por endometriosis?

o Sí (1)

o No (2)

¿Cree que hay suficientes recursos disponibles para usted para manejar la endometriosis?

o Sí (1)

o No (2)

o No estoy seguro (4)

¿Hay algo más que le gustaría compartir sobre su experiencia al acceder a la atención por endometriosis?

o Sí (Respuesta Abierta) (1) __________________________________________________

o No (2)

End of Block: Perception of Care - Spanish

Start of Block: Conclusion - Spanish

Gracias por tomarse el tiempo para completar esta encuesta. Su aporte es invaluable para ayudarnos a mejorar el acceso a la atención para los pacientes con endometriosis. Si tiene alguna pregunta o comentarios adicionales, no dude en ponerse en contacto con nuestro equipo de investigación: Eliana Burgos, MS2 (Elianaburgos@usf.edu)

End of Block: Conclusion - Spanish
